# In Silico Conotoxin Studies: Progress and Prospects

**DOI:** 10.3390/molecules29246061

**Published:** 2024-12-23

**Authors:** Ruihan Li, Md. Mahadhi Hasan, Dan Wang

**Affiliations:** 1 Department of Chinese Medicine and Pharmacy, School of Pharmacy, Jiangsu University, Zhenjiang 212013, China; 3211603004@stmail.ujs.edu.cn; 2 Division of Chemistry and Structural Biology, Institute for Molecular Bioscience, The University of Queensland, Brisbane, QLD 4072, Australia; mahadhi@pharm.ku.ac.bd; 3 Pharmacy Discipline, Life Science School, Khulna University, Khulna 9208, Bangladesh

**Keywords:** venom peptides, in silico strategies, drug discovery, binding modes

## Abstract

Cone snails of the genus *Conus* have evolved to produce structurally distinct and functionally diverse venom peptides for defensive and predatory purposes. This nature-devised delicacy enlightened drug discovery and for decades, the bioactive cone snail venom peptides, known as conotoxins, have been widely explored for their therapeutic potential, yet we know very little about them. With the augmentation of computational algorithms from the realms of bioinformatics and machine learning, in silico strategies have made substantial contributions to facilitate conotoxin studies although still with certain limitations. In this review, we made a bibliometric analysis of in silico conotoxin studies from 2004 to 2024 and then discussed in silico strategies to not only efficiently classify conotoxin superfamilies but also speed up drug discovery from conotoxins, reveal binding modes of known conotoxin–ion channel interactions at a microscopic level and relate the mechanisms of ion channel modulation to its underlying molecular structure. We summarized the current progress of studies in this field and gave an outlook on prospects.

## 1. Introduction

Over the centuries, venomous animals have evolved biodiverse venom toxins for defensive and predatory purposes. As a thriving phylum in tropical marine habitats, cone snails exhibit an exceptional range of venom diversity, each one producing a distinct venom, containing more than 200 individual venom peptides called conotoxins (or conopeptides) [1,2]. Conotoxins have been defined as “disulfide-rich” conopeptides, but it was later proven inappropriate to distinguish between “disulfide-rich” and “disulfide-poor” conopeptides [3]. It is estimated that there are over one million different biologically active conotoxins, yet over 99% of them remain to be sequenced [1,4]. The underlying reason for the structural diversity of conotoxins is the requirement of improving defense and predation capabilities to adapt to millions of years of positive selection in highly competitive races [5]. Conotoxins are small peptides, typically consisting of 10–45 amino acids, but larger proteins have been discovered recently [6,7]. These toxins are tightly packed by disulfide bonds, forming highly stable and ordered loop structures and unique bioactive folds, the protein-like secondary motifs of α helices, β turns, and β sheets. It is their disulfide bond frameworks that allow for high potency, refined receptor subtype selectivity, and resistance to proteases [4,8]. In addition to disulfide bonds, cone snail peptides frequently carry a variety of post-translational modifications (PTMs), and up to 75% of amino acids in a single conotoxin are post-translationally modified [9,10,11], including hydroxylation of proline and valine, bromination of tryptophan, gamma-carboxylation of glutamate, C-terminal amidation, and pyroglutamation [12]. The aa number and sequence, secondary structures, and PTMs make the biodiversity of conotoxins possible.

Recent years have witnessed the availability and refinement of computational resources and algorithms, and machine learning (ML) is playing an increasingly important role in drug discovery. Drug discovery is a complex and time-consuming process involving validating the target, discovering drug leads that interact with the target, evaluating the safety and efficacy of the drug leads through preclinical studies and clinical trials, and ultimately obtaining new drug approval. Whereas traditional methods often require a lot of experimentation and high expense, ML can take advantage of a plethora of high-dimensional complex data and algorithms to speed up the drug discovery process and improve efficiency and accuracy [13]. ML algorithms, such as Random Forest (RF), Naive Bayesian (NB), support vector machine (SVM), etc., have been used to develop new uses for drugs, discover new therapeutic effects of drugs, predict drug–target interactions, ensure safe biomarkers, and optimize the biological activity of the molecule [14,15,16,17,18]. According to many in this field, NB contributes to decreasing computational complexity and time consumption by ranking molecular descriptors based on their importance and selecting the most discriminative features. The NB method effectively deals with the high-dimensional data space created by the use of many molecular descriptors for compound characterization in drug discovery, allowing researchers to efficiently screen drug candidates with potential pharmacological activity [19]. Whereas NB makes good use of molecular descriptors, the use of RF can improve the selection of molecular descriptors. In RF, an ensemble of multiple decision trees is constructed to better predict the activity or classification of a molecule through voting or averaging the prediction results [20]. Comparatively, SVM, another widely used ML method, is thought to be essential to novel drug discovery since it has the capability to distinguish between active and inactive compounds, construct regression models and rank (virtual screen) data-based compounds [21,22].

Except from ML, computational chemistry methods, like molecular docking and Molecular dynamics simulations are also making essential contributions to the field of Bioinformatics and Proteomics [23]. Docking strategies provide in silico simulations of the interactions between receptor and ligands, predict ligand–receptor binding energy and affinities, and can be used for high-throughput drug screening. In molecular docking of a peptide ligand to its target, a 3D grid was constructed around the identified active sites to calculate the binding energy between the ligand and the protein after pre-processing the 3D structural information of proteins and ligands sourced from the Protein Data Bank (PDB) database, and the docking result was determined based on the score and the conformation or position of the ligand with the highest binding affinity [24,25,26]. Docking performance of non-small molecule ligands to a target protein, in general, is still limited by some nonnegligible problems, including how to stimulate flexibility of the target binding site, how to assess protein–protein interactions, and how to deal with the conformational changes in the target protein during the process of ligand binding [26,27].

In this review, the increasing use of in silico strategies in conotoxin studies over the last two decades was highlighted through a bibliometric analysis. We then briefly summarized the three main classification approaches of conotoxins and introduced the applications of machine learning methods in conotoxin classification, which not only aid in the isolation and identification of conotoxin sequences and structures but also hold great significance in accelerating novel conotoxin drug discovery. We discussed the uncovered modulation mechanisms and binding modes of conotoxins to their targets at the molecular level achieved through computer-aided approaches and proposed the current drawbacks of the field and the potential research prospects in the future.

## 2. Bibliometric Analysis of In Silico Conotoxin Studies

### 2.1. Data Collection

Recent developments in the field of in silico drug discovery from venom toxins have sparked considerable interest in studies on conotoxins for their diversity and selective bioactivity, here, we provide bibliometrics data of the literature on in silico conotoxin studies ranging from 2004 to 2024 to fill in the bibliometric analysis gap of in silico conotoxin research hotspots and trends. The information was retrieved from the Web of Science Core Collection on 4 August 2024, using the query “TS = (“conotoxin$” or “conopeptide$” or “toxin from cone snail$” or “toxin$ from cone snail” or “venom toxin from cone snail$” or “venom toxin$ from cone snail” or “venom from cone snail$” or “venom peptide from cone snail$” or “venom peptide$ from cone snail” or “peptide toxin$ from cone snail” or “peptide toxin from cone snail$”) AND TS = (“machine learning” or “deep learning” or “computer aided drug design” or “computer-aided drug design” or “in silico” or “computational studies” or “computational method$” or “computational approach*” or “artificial intelligence” or “algorithm$” or “molecular docking” or “docking” or “molecular dynamics” or “homology modeling” or “Linear Regression” or “Random Forest” or “Support Vector Machine” or “K-Nearest Neighbors” or “Decision Tree” or “K-Means” or “Naive Bayes” or “Dimensional Reduction” or “Gradient Boosting” or “Logistic Regression”)”. A total of 282 articles and reviews were searched through the combination of single-topic retrieval and multi-topic retrieval. The obtained data were then screened in CiteSpace for repetition and inconsistency with the topic, and eventually, 271 valid documents were exported with full records and cited references in plain text format, involving 234 articles, 32 reviews, 4 proceedings papers, and one book chapter. These documents came from 977 authors from 603 institutions in 102 countries or regions and were published in 114 journals.

### 2.2. Data Analysis

**Figure 1:**
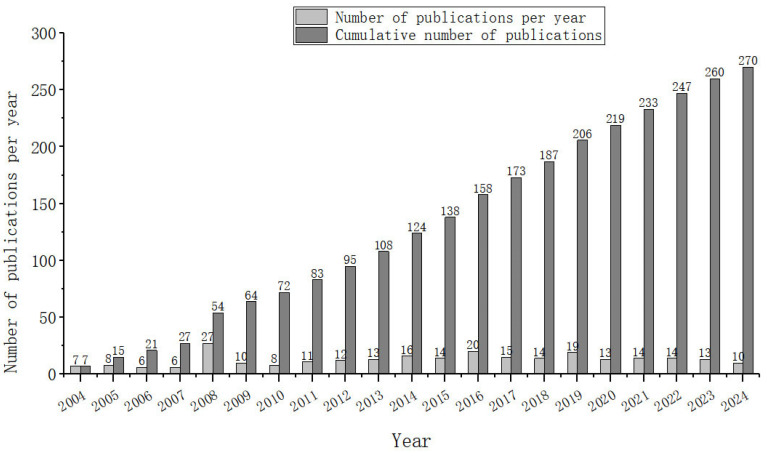
Year-by-year changes in the number of published documents on in silico conotoxin studies in the period from 2004 to 2024.

Figure 1 illustrates the temporal distribution and changes in research results published in the field. Generally, the cumulative number of publications is rising, and the number of publications per year is tending towards stability, which indicates that constant attention has been paid to this field in recent years.

**Figure 2:**
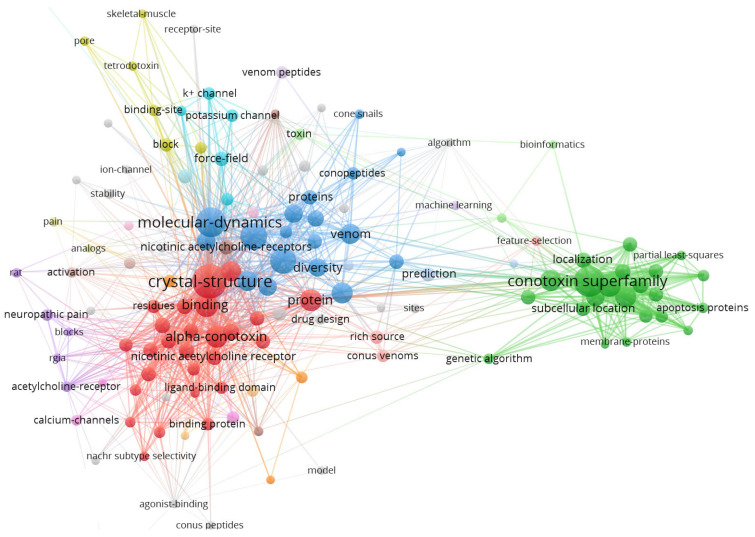
The keyword co-occurrence of bibliometric analysis of in silico conotoxin studies from 2004 to 2024 made by VOSviewer. Some parameters in this figure are listed as follows: Type of analysis—Co-occurrence; Unit of analysis—All keywords; Counting method—Full counting; Minimum number of occurrences of a keyword—5. The normalization method is association strength. The layout is set with Attraction = 2 and Repulsion = 0. Among the 1560 keywords, 134 meet the threshold.

After the milestone of ziconotide in 2004, the first peptide toxin drug approved by the Food and Drug Administration (FDA), the keyword trends in the conopeptide literature changed in the wake of research hotspots transferring from a research focus on analgesic drug discovery on voltage-gated calcium channel that eventually led to the success of ziconotide to a broader pharmacological characterization of conopeptides [28], and our keyword co-occurrence analysis of in silico conotoxin studies from 2004 to 2024 further confirmed a broad range of research hotspots with multiple connections (Figure 2). According to frequency (Table 1), apart from “conotoxin”, the top three keywords were “crystal structure”, “conotoxin superfamily” and “molecular dynamics”. The crystal structures were discussed most frequently with 50 occurrences. The importance of crystal structures is nonnegligible as they provide precise details on the three-dimensional (3D) arrangement of atoms within protein structures, which are essential for understanding the mechanism of their function and serve as starting points for molecular dynamics simulations. Moreover, structures of conotoxins in complex with their molecular targets accurately reveal drug–target interactions and provide indispensable tools for in silico conotoxin drug discovery [29,30]. Studies on conotoxin classification are highly bioinformatics dependent, the complex disulfide bond patterns and 3D structures of conotoxins present a challenge for in silico modeling, but the classification of superfamily makes it more convenient to identify their precursor signal peptides and organize the vast number of conotoxin sequences [31]. Therefore, the keyword “conotoxin superfamily” is not surprisingly ranking high in occurrence. Molecular dynamics plays a pivotal role in in silico conotoxin studies, providing detailed insights into the structural and dynamic properties of these bioactive peptides. Researchers have studied conformational changes and stability under the influence of the oscillating electric fields and found that the applied oscillating electric field of 4.7 × 10^−8^ V/nm produced changes in the conformation of conotoxin [32,33].

**Figure 3:**
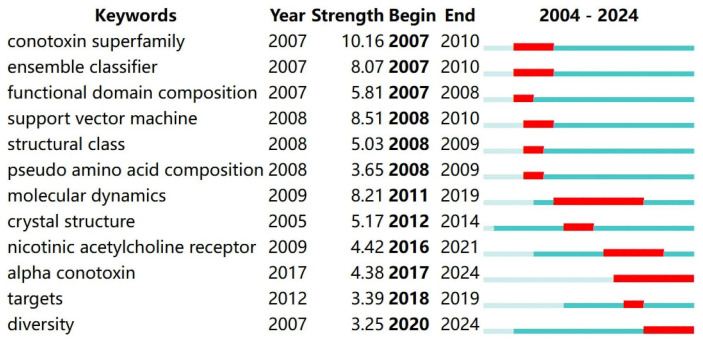
Top 12 keywords with strong citation bursts of in silico conotoxin studies from 2004 to 2024 made by CiteSpace. Some parameters used are listed as follows: Term Source—Title, Abstract and Author Keywords; Node Types—Keyword. The detection model is configured with γ = 1.0 and Minimum duration = 2, resulting in 12 burst items found. Some labels in the figure require to be explained: The blue lines indicate the period when the keywords were used and the red ones represent the burst periods. The “Strength” column reveals the frequency of each keyword. The higher the strength, the more popular the corresponding keyword.

In addition to the keyword co-occurrence analysis, a burst keywords analysis was also performed to demonstrate the research trends in a particular period of time and research frontiers in recent years, as well as suggesting that of the future [34]. The twelve discovered burst keywords are shown in Figure 3, among which “support vector machine”, “pseudo amino acid composition”, “molecular dynamics” and “nicotinic acetylcholine receptor” are also indicated as burst keywords in the broader field of conopeptide studies during the period 2000–2022 [28]. The period between 2007 and 2010 witnessed a significant increase in interest in computational methods, particularly in ‘ensemble classifier’ and ‘support vector machine’, indicating a key turning point towards utilizing advanced computational techniques for conotoxin classification and analysis. In the period (2007–2009), the focus on “functional domain composition” and “structural class” suggests an early emphasis on understanding the molecular structural and functional roles of conotoxins, laying foundational knowledge for subsequent research. Subsequently, the sustained period of interest in “molecular dynamics” and “crystal structure” from 2009 to 2019 underscores a long-term commitment to studying the dynamic behavior and structural properties of conotoxins, which is essential for deciphering their mechanisms of action. It is worth mentioning that the year 2005, when “crystal structure” became a keyword in in silico conotoxin studies, is a watershed for the first crystal structure of eukaryotic voltage-gated ion channel (K^+^ channel) and the refined structure of Nicotinic Acetylcholine Receptor to be reported [35,36]. The citation strength of “nicotinic acetylcholine receptor” from 2009 to 2021 highlights a milestone in exploring the interaction of conotoxins with neurological targets, a critical aspect of their potential therapeutic use. The term “targets” (2012–2019) experienced a citation burst, indicating a concerted effort to identify and understand the specific molecular targets of conotoxins, a key step in drug development. From 2007 to 2024, the consistent interest in “diversity” over the years underscores the recognition of the vast array of conotoxins and the exploration of their evolutionary and functional significance. In addition, the recent surge in “alpha conotoxin” research from 2017 to 2024 suggests a refinement of focus on specific conotoxin subtypes, which may offer unique therapeutic opportunities.

To some extent, the in silico field of conotoxin studies has been highly active and attracted continuous and considerable attention in the past 20 years. However, machine learning algorithms have not been widely applied to polypeptide prediction mainly due to structure limitations. We expect that the current bibliometric analysis can provide directions to the application of in silico strategies, inspire innovative perspectives and set the stage for future discoveries.

## 3. In Silico Strategies in Conotoxin Classification

Recent advancements in venomics, especially 454 pyrosequencing, mass spectrometry, and Sanger sequencing technologies, have proven useful in expanding our knowledge of venom peptides and accelerating new discoveries [37,38,39]. Early studies focused on the exploration of intraspecific variations at the species level, including *Conus bayani* [40], *Conus betulinus* [38], *Conus striatus* [41], etc. In a recent study, Zheng et al. sequenced the transcriptomes of 34 cone snail species from the NCBI databases and assembled the transcripts of each species, identifying a total of 4111 conotoxins. It is, therefore, not surprising that the complexity of Conus venom peptides was acknowledged [42], and the vast existence of PTMs provides a new level of diversity [43].

Although conotoxins exhibit an extensive array of chemical diversity with the presence of more than 1000 different peptides in a single venom [1,44,45], certain similarities do emerge, and it is customary to employ the following characteristics for classification: (i) the commonalities between the Endoplasmic reticulum (ER) signal sequence of the conotoxin precursors (gene superfamilies); (ii) a characteristic arrangement of cysteine residues in the mature peptide regions of conotoxins (independent of their connectivity) (cysteine frameworks); (iii) interactions between the conotoxin and its molecular target (pharmacological families) [46]. In the following sections, we will briefly introduce conotoxin classification approaches list their detailed classification, and how computational strategies can be used to understand and predict the structure and function of conotoxins.

### 3.1. Three Primary Approaches for the Classifications of Conotoxins

#### 3.1.1. Gene Superfamilies

Conotoxin precursors are the initial translation products and typically have three well-defined regions consisting of the *N*-terminal signal sequence, followed by a “pro” region then the “toxin” region that encodes a single copy of the mature peptide near the *C*-terminus [47]. The “pro” peptide is thought to play a role in conotoxin maturation and is removed prior to secretion of the mature conotoxin peptide. The mature peptide region has evolved rapidly and exhibits high diversity, while the endoplasmic reticulum signal sequence has remained more conserved. Conotoxins are grouped into gene superfamilies according to similarities in the signal sequences of conotoxin precursors [26,48,49]. Currently, conotoxins can be classified into 29 major superfamilies: A, B1, B2, B3, C, D, E, F, G, H, I1, I2, I3, J, K, L, M, N, O1, O2, O3, P, Q, R, S, T, U, V, Y [46].

#### 3.1.2. Cysteine Frameworks

A key feature of conotoxins is their highly conserved cysteine framework [7], which is distinguished by the number and arrangement of cysteine residues along the sequence, distinct disulfide bond connectivity, and their loop size based on each gene superfamily [49,50]. To date, 32 framework families have been sorted in ConoServer. Interestingly, mature regions can contain variable amounts of cysteines (4, 6, 8, 10, 12), and their respective positions are not fixed in place, for instance, four cysteines can be arranged as the cysteine pattern of CC–C–C or CC–CC or CC–C–C or C–C–C–C or C–C–CC or C–CC–C, where ‘–’ represents a variable number of amino acids. The connectivity of the disulfide bonds, in the last column of Table 2, is explained by previous studies in the following way: during the maturation process, certain toxin residues undergo PTM, of which the important and most prevalent formation is the disulfide bonds [51,52]. Disulfide bonds could stabilize the protein-like secondary structure and form a unique bioactive fold. The tight packing of these toxins by multiple disulfide bonds significantly influences their structural definition [8,53]. Conotoxins with similar cysteine frameworks may exhibit different disulfide connectivity, resulting in vastly different structures. For example, EpI and TIA share a similar cysteine framework I within gene superfamily A but demonstrate distinct structural and functional differences due to different disulfide bond connectivity, representing alpha conotoxin and rho conotoxin pharmacological families, respectively.

#### 3.1.3. Pharmacological Families

Previous experimental studies have shown that conopeptides with clinical potential are primarily characterized pharmacologically after their sequences have been determined [56,57] According to the explanation in ConoServer, conopeptides are grouped into 12 pharmacological families based on their target receptor (ion channels and transporters) and the type of interaction with the receptor (Table 3) [4].

### 3.2. Conotoxin Classification by Using Machine Learning Methods

A considerable number of published studies have described the diversity of structure and pharmacology characteristics of conotoxins. Conotoxins are believed to be excellent pharmacological probes and potential drug candidates, especially for neurological drug design and development [58], as their therapeutic potential is mainly associated with particular targets in the nervous system [59]. As conotoxin has undeniably become a research hotspot, and with the improvement in sequencing techniques, the number of conotoxin sequences is increasing exponentially, calling for automating superfamily elucidation.

#### 3.2.1. The Construction of Benchmark Datasets

In the process of protein classification with machine learning methods, the first step is to construct a reliable benchmark dataset to preprocess the samples of conotoxins from the general databases or the specialized ones [60,61,62,63]. The database specifically built for conotoxins is called ConoServer, created by the lab of David Craik in 2007 [54]. ConoServer involves the sequence, structure, genetic, and pharmacological information of currently available conotoxins, which sources from the peer-reviewed literature as well as the publicly available databases, involving UniProtKB/Swiss-Prot, NCBI nucleotide, and the Worldwide Protein Data Bank.

#### 3.2.2. The Description of Conopeptide Samples

After the construction of the dataset, the peptide toxin samples need proper description involving two strategies: the continuous model and the discrete model. As sequence similarity is required for the continuous model, the discrete model is more recommended for formulating conotoxin samples [64,65]. Pseudo amino acid composition (PseAAC) [66], which represents a protein sequence in a discrete model without completely losing its sequence-order information, is currently the most popular method for predicting protein structural and functional attributes and is widely used in conotoxins classification [67,68]. Wu et al. used PseAAC to formulate samples to identify different types of ion channel-targeting conotoxins [69]. Once the benchmark datasets and protein samples are constructed, the subsequent problem is how to find an effective prediction engine to train them and conduct the predictions [70].

#### 3.2.3. Predict Conotoxin Superfamilies Efficiently Through Machine Learning

The encouraging results, as shown in the literature [50] indicate that many methods proposed to predict conotoxin superfamilies perform well, particularly multi-class SVM. It outperforms common methods like BLAST, ISort, and distance-based predictors, achieving an 88.1% accuracy for predicting A, M, O, and T superfamilies through jackknife cross-validation [65]. Continuous efforts have been made to further improve prediction accuracy and scope, the studies of Zaki et al., as an example, set out to enhance the degree of exactitude and combine SVM with Freescore based on local alignment partition functions. The approach, SVM-Freescore, quantifies the similarity between protein sequences, presenting them as feature vectors, to achieve the ultimate purpose of accurately classifying conotoxin gene superfamilies [70]. The increasing development of sequence techniques leads to a challenging amount and complexity of precursor structures [71,72], which require methods that have the ability to deal with complicated biological problems. Towards this end, Fanet al. proposed a novel technique called PredCSF to predict superfamilies directly from the amino acid sequence, which mixes multifarious sequential characteristics [31]. Despite its high performance, SVM is always facing an inevitable shortcoming of extensive training requirements [73]. To conquer this trouble, Johnson et al. developed a search heuristic, HMMERHEAD, and made prominent contributions to shorten the time for scoring profile hidden Markov models (PHMMs) based on huge sequence databases, while detecting more remote protein homologs [74]. Besides enormous training demands, the high comparability of protein sequences has also become a stumbling block to predicting classifications precisely. To deal with this problem, two studies tried to integrate PHMMs with Position-Specific Scoring Matrices (PSSMs), and build an online tool called ConoDictor that enables fast and accurate classification of conotoxins into superfamilies based on their amino acid sequence [75,76,77]. In 2012, Laht et al. established 62 PHMMs to identify all conopeptide superfamilies, and surprisingly, the accuracies of mature peptide, the pro-, and signal peptide models are, respectively, 96%, 100%, and 100%. To be noted, this was the first success in predicting all known gene superfamilies of conotoxins with Machine Learning [77]. Lavergne et al. described an algorithm named ConoSorter that categorizes cDNA or protein sequences into conotoxin superfamilies and classes and differentiates known and novel conotoxins through automatic access to the ConoServer database for known precursor sequences. They successfully identified 158 novel precursor conopeptide transcripts, 106 of which were confirmed by protein mass spectrometry [78], and identified 13 novel conotoxin gene superfamilies, illustrating its application not only in conotoxin gene superfamily prediction but also in de novo conotoxin identification.

## 4. In Silico Drug Discovery from Conotoxins

### 4.1. Quantitative Structure–Activity Relationships

Quantitative structure–activity relationship (QSAR) modeling is one of the most popular in silico approaches, where the quantitative numerical measure of chemical structures (e.g., physiochemical parameters) are statistically related to their physical properties (e.g., charges) or biological activities [79,80,81,82], and could uncover the mechanism of action in the meantime [83]. For example, in 2009, Khoo et al. revealed the crucial residues of μ-conotoxin KIIIA for the inhibition of Na_V_1.2 and Na_V_1.4 with the help of QSAR analysis [84]. QSAR modeling could also be a powerful assist in memetics design by providing chemical descriptors useful for filtering combinatorial libraries and compound collections. In conotoxin studies, mutated or synthetic conotoxin structures could be predicted in QSAR models [4].

A considerable amount of studies have demonstrated that computer modeling-assisted analysis (CMAS) has aided in determining structure–activity relationships of native peptides [79], among which the Gaussian processes technique was applied to the classification of QSAR modeling [85], and comparative interaction fingerprint analysis (CoIFA) assist in constructing the QSAR model [86]. As a top-performing methodology, peptide QSAR studies are now in wide application, and it is suggested that the ML-based QSAR model currently plays a significant role in drug design and virtual screening, property and classification prediction, etc. [87,88]. Liu et al. took into account the probable distinct performance of QSAR prediction of domain–peptide interactions with four sophisticated machine learning models (MLMs), including one linear partial least squares (PLS) and three nonlinear MLMs, including RF, SVM and Gaussian process (GP). The results suggested that although the nonlinear GP, RF and SVM gave stronger fitting on the training set than linear PLS, all four approaches showed similar predictability on the test set [87].

### 4.2. Cone Snail Multi-Omics Integration

Although PCR and gene cloning are probably practical with one or few specimens, they are inferior to the transcriptomic and proteomic approaches for being relatively low throughput, sample and time-consuming. To efficiently decipher conotoxin transcriptomes and proteomes, NGS platforms, such as 454 (Roche, Branford, CT, USA) and Illumina (Illumina, San Diego, CA, USA), have been used [89,90]. The development of a high-resolution mass spectrometry instrument [91] with an advanced mass analyzer (TOF) and efficient ionization technique (ESI), also facilitated the identification of mature peptides [92]. For instance, the venom of *C. marmoreus* was comprehensively analyzed by mass spectrometry (MS), with 2710 and 3172 peptides detected utilizing matrix-assisted laser desorption ionization (MALDI) and ESI-MS, respectively, and 6254 peptides using an ESI-MS TripleTOF 5600 instrument [1]. In 2017, Gao et al. screened out six conotoxins with potential insecticidal activity from their comprehensive library made up of 215 conotoxin transcripts through a homological search with a reported positive control of α-conotoxin ImI as the query [93]. Inspiringly, this process can be accomplished more effectively and precisely through newly developed in silico tools, such as ConoSorter and ConusPipe [78,94,95].

Both transcriptomics and proteomics independently produce a significant number of novel conopeptides, with an overlap and march rate of only 9.98% [96]. Thanks to the complementary cumulative effect of transcriptomics and proteomics, these big-data-based approaches are integrated to reveal an increasing number of novel venom peptides, meanwhile, providing new perspectives to the important and complicated venom biology. Nevertheless, the goal of novel conotoxin discovery extends beyond this point and eventually aims to discover and validate pharmacologically active venom peptides, which also require a high-performance tool to accomplish massive data processing and integration [9,39,97].

### 4.3. Prediction of Common Targets and Binding Site with Modulators

#### 4.3.1. Conotoxins Targeting Voltage-Gated Ion Channels

The screening for venom peptide modulators of voltage-gated ion channels (VGIC) has long been a focused area in drug discovery, especially in the discovery of new neurological therapeutics. VGIC are integral membrane proteins that play diverse physiological roles and function in many aspects of neurotransmission, as well as participating in the modulation of various cellular processes. There are three members of gene superfamilies encoding VGIC, including voltage-gated potassium channels (VGPC), sodium channels (VGSC) and calcium channels (VGCC). They are key players in cellular excitability and are targets for many pharmacologically valuable venom toxins and medically important drugs. Selective venom toxin modulators for VGIC provide unique probes for ion channel structure-function and potential new drug leads for the treatment of a wide range of pathophysiological conditions, including neurological disorders and diseases, cancers, and inflammation. In recent years, with the rapid development of structural biotechniques, notably cryo-electron microscopy (Cryo-EM), eukaryotic VGIC structures have been largely revealed, which encouraged the use of computational technologies in faster drug screening of better pharmacological tools and therapeutic leads. While the first structure of eukaryotic VGPC (K_V_1.2) was reported back in 2005 [36], it was not until 2015 [98,99] and 2017 [100], respectively, for the first eukaryotic VGCC (Ca_V_1.1) and the VGSC (Na_V_PaS) structure to be published.

As natural ligands for the nervous system, conotoxins have been widely explored for their therapeutic potential through targeting the VGIC. The clinical success of conotoxins start from FDA approval of ziconotide (Prialt^®^) [9], the first marine-derived nonopioid analgesic. Ziconotide precludes presynaptic neurotransmitters from releasing through selectively blocking N-type VGCC, for the treatment of severe chronic pain conditions [8]. Thereafter, more conotoxins have emerged as drug candidates for analgesic development, like μ-conotoxin BulllB (μ-BulllB) [101], μ-GllA, μ-GllB [102], and χ-MrlA [103]. These toxin modulators represent new analgesic mechanisms through interaction with VGIC and provide new market opportunities. Machine learning techniques have been applied to the precise prediction of ion channel types as potential targets for conotoxins, and several approaches have been developed for the prediction, as shown in Table 4.

Table 4 summarizes the overall and average accuracy of different algorithms for the prediction of conotoxin–ion channel interactions. Among them, the F-score–SVM method outperforms the others with an overall accuracy of 94.6%, and it makes strong predictions over imbalanced datasets. The AVC–SVM (Analysis of Variance and Correlation–Support Vector Machine) algorithm, achieving an overall accuracy of 91.98%, is effective in the classification of biological data. IonchanPred 2.0, designed specifically for ion channel prediction, gives an overall accuracy of 92.6% but a lower average accuracy of 87.7%. The RBF (Radial Basis Function) network3, with an overall accuracy of 89.3%, is effective in dealing with non-linear data. The predictor of iCTX-Type performs well in the categorization of ion channels-targeted conotoxins, and so is the random forest-based predictor ICTCPred. Future studies should focus on refining these models and exploring hybrid approaches to improve prediction accuracy further.

#### 4.3.2. The Mechanisms of Toxin-VGIC Modulation

A plethora of natural polypeptides, representatively the conotoxins, are devised to be promising lead compounds of antiepileptics, antiarrhythmics, and analgesics through modulation of VGIC (Table 5). According to the mechanisms of action, the modulators are sorted into two groups: pore-blockers and gating modifiers (or allosteric modulators) [8,109].

The pore blockers engage with the ion channel vestibule and block the ion influx via specific VGIC subtypes depending on their affinity and selectivity. More vividly, conotoxins are regarded as “lock-and-key” inhibitors that bind to and change the protein structural and functional characteristics of their target [8,26,88,109]. Numerous studies have attempted to make the mechanisms of pore blockers fully understood. In 2011, relying on in silico modeling and functional testing approaches, the structure of the KIIIA-hNa_V_1.7 complex was predicted. The results indicated that µ-conotoxin KIIIA bound to the upper region of the selectivity filter and compressed open space, blocking Na_V_1.7 incompletely [110]. In 2023, Kimball et al. employed an in silico alanine scan to identify the key residues in KIIIA binding to Na_V_1.7 on the channel surface using Rosetta computational modeling combined with RosettaDock [111]. Their analysis successfully revealed key residues of K7, W8, R10, H12, and R14 in the binding energy level, forming salt bridges and hydrogen bonds [111]. There have been similar studies on µ-conotoxins PIIIA, SIIIA, and GIIIA, predicting their interactions with K_V_ or Na_V_ channels [112,113].

In silico studies of pore blockers can be generalized into three steps, starting with predicting binding modes between toxin and channel, with molecular docking and molecular dynamics simulations [8]. Computational modeling will then be used to determine the key interacting amino acid residues in a specific toxin or in its targeting channel, so as to calculate the corresponding binding affinity [114]. When the structures of channels are unattainable, it is a practical way to construct a homology model for the pore domain of the VGIC, which is now getting easier with the development of AlphaFold, an AI system that predicts protein 3D structure from amino acid sequence. Interestingly, the model can also be employed as a template in constructing homology models for other channels of the same family due to high homology [8]. Eventually, experimentally observable parameters are calculated, like relative or absolute free energy, interaction energy, etc., to validate the predicted binding modes, ruling out the unrealistic or incorrect ones [8,114,115].

Docking studies and molecular dynamics complement each other and provide in silico representation of dynamical conformations and binding states of toxin–target interactions at a microscopic level, grounding on 3D structures of toxin–target complexes [24]. Molecular dynamics simulations, which are solving problems using Newton’s laws of motion and the velocity Verlet algorithms, can work out conformational entropy and predict dynamical fluctuations, as well as present binding sites and ligand–receptor interactions [8,26,116]. Two popular approaches are the thermodynamic integration [117] and umbrella sampling [118], which aim to describe potentials of mean force (PFM) and determine free energy. For instance, Chen et al. validated the binding modes of two pore blockers with their targets, GVIA–Ca_V_2.2 and PIIIA–Na_V_1.4 complex with molecular dynamics simulations, respectively, in 2013 and 2014 [119,120]. Additionally, in the study of PIIIA–Na_V_1.4 binding simulation, their binding affinity (Kd) are accurately computed, with an acceptable range of differences from experimental values [120,121,122].

Unlike pore blockers, which spatially block the conductive pathways of the channels, gating modifiers alter the conformational states of the channels by interacting with one or more voltage-sensing domains of channels, achieving effect on the dynamic characteristics and gating of the channels [8,123,124]. In other words, the modulations of gating modifiers can be accomplished through conversion between activation and inactivation gating of ion channels, inducing or preventing the entrance of sodium or calcium ions into the cell, for the adjustment of neuronal excitability and perception of neuropathic pain [8,125].

δ-conotoxins are known to participate in the fast inactivation of Na_V_ channels [126]. However, in our previous study, δ-conotoxin TxVIA, as a gating modifier of the mollusc Na_V_ channels, was confirmed to be ineffective on human Na_V_ channels. Instead, it showed an activating effect on human Ca_V_3.1, and surprisingly, showed inhibitory effect on human Ca_V_3.2. We also predicted the binding mode of TxVIA with Ca_V_3.x using human Ca_V_3.1 Cryo-EM structure and homology models of Ca_V_3.2 and Ca_V_3.3 [127], results of which showed high affinity of TxVIA binding to DIV S3-S4 linker of Ca_V_3.1. It is noteworthy that Rocio K. et al. conducted a docking study of PIIIA with NaChBac, which revealed that the two mentioned binding modes could happen on PIIIA. In addition to obstructing the channel-mediated currents by directly impeding the conducting pathway, PIIIA may also influence the relative proportions of conducting (activated) and non-conducting (inactivated) states [128].

#### 4.3.3. Conotoxins Targeting Nicotinic Acetylcholine Receptors

nAChRs are pentameric ligand-gated ion channels from the Cys-loop superfamily, and are widely distributed in the central and peripheral nervous systems. nAChRs play pivotal roles in the nervous and motor systems through mediation of the neurotransmitter acetylcholine, and their modulators have been explored for therapeutic potential in the treatment of neurological disorders and diseases, such as Alzheimer’s disease, Parkinson’s disease, and pain [129,130,131]. Each subunit of nAChRs is divided into three domains: an extracellular domain (ECD) responsible for ligand binding, a transmembrane domain (TMD) consisting of four transmembrane helices (TM1-4), and an intracellular domain (ICD) [129,132,133]. In 2001, Brejc et al. reported the first crystal structure of the acetylcholine binding protein (AChBP) [35], which marks the first milestone in the in silico studies on nAChRs. However, the lack of high-resolution experimental structures of nAChRs has been a barrier to computational studies [131,134,135], which was then solved by the ground-breaking work of Unwin et al. [133] in 2005. They reported the refined Cryo-EM structures of a Torpedo acetylcholine (ACh) receptor of unliganded closed state at 4 Å resolution and of Ach liganded open state at 6.2 Å resolution, which have been considered the most important milestones in the affirmation of conotoxin action modes. To a great extent, the resolution of complex models affects the prediction accuracy of interactions between ligands and receptors [131]. In more recent studies, the analogical computational toxin-AChBP binding models have been reported, such as α-conotoxin RgIA, RgIA4 [136], ImI [137], PeIA [134], LvIA [138], GID [135], and their analogs in complex with AChBP, providing diversified tools for in silico conotoxin studies.

Retrievable algorithms to refine conotoxin–nAChRs complexes are listed as follows in chronological order. In 2011 [139], Yu and colleagues refined the structures of 16 mutated α-conotoxin ImI/α7nAChR complexes with the minimization-based approach (MBA) or the molecular dynamics simulation-based approach (MDBA), and results with MBA reached better consistency between computational models and experimental values [139]. Leffler and Kuryatov et al. [135] developed a docking algorithm called ToxDock. ToxDock applies ensemble-docking and extensive conformational sampling to dock conotoxin and their analogs to nAChR homology models and refine models of these complexes by integrating two existing protocols, Rosetta FastRelax and Rosetta FlexPepDock [111]. This study was in the context of docking α-conotoxin GID to the homology model of α4β2 nAChR, but the application of ToxDock is certainly extensible to other peptide toxins and receptors. Energy minimization (EM) and molecular dynamics simulations were also attempted in structure refinement, and are believed to refine the conformation of the a7–nAChR/ImI model significantly [134,139].

In general, in silico approaches for investigations of conotoxin–nAChR interactions associated with novel drug discovery have mainly been achieved by using molecular dynamics (MD) and molecular docking. As an example, MD proved that Ser-9 of TxID is bound to α6β4 nAChR through a weak hydrogen bond with β4 Lys-81 [140], and the model demonstrated the binding pocket, where Met-11 of TxID was encompassed by hydrophobic and charged amino acids, including Ile78, Ile110, Arg112 and Arg80 [141]. With MD strategies, key residues in the binding modes have been largely uncovered [136,142,143,144,145,146]. Docking and MD simulations could also compensate each other in analysis, like in the study by Li et al., the application of docking and MD successfully revealed that Glu197 in the α10 subunit, Asp168 in the α9 subunit and Asp205 in the α10 subunit participated in the RgIA-α9α10 nAChR interaction [147].

Computational modeling could also be used to guide experimental characterization [136,148,149], with successful achievements in rational mutagenesis of conotoxins, GeXIVA [150], TxIB [151], and ImI [151] following predicted enhancing or declining, or even conversing binding affinities. In a recent study by Li and Tae et al., Dab/Dap-substituted analogues of α-conotoxin PeIA were synthesized, and the most potent mutant PeIA[S4Dap, S9Dap] was revealed in MD simulations to have adopted extra hydrogen bonds and electrostatic interactions between Dap and residues at the α9(+)-α9(−) binding site of human α9β10 nAChRs [142].

Virtual screening has been playing significant roles in novel drug discovery. Although the currently available virtual screening strategies are generally not applicable to the screening of peptide drugs, attempts have been made at conotoxin drug discovery by targeting nAChRs. As an example, an algorithm named Genetic Algorithm Managed Peptide Mutant Screening (GAMPMS) was developed in 2016 to conduct high throughput virtual screening on enormous mutant libraries of α-conotoxins, searching for conotoxins with greater binding affinities to nAChR subtypes [151,152]. Similarly, the Fold server [153] and ToxDock [135] also gave fantastic performances in predicting qualified conotoxins binding to nAChRs. Unlike experimental high-throughput screening of conotoxin ligands for nAChRs, which are always restricted by high expense, high time cost, and non-universal applicability for constructing large libraries, virtual screening provides efficient design of large conotoxin mutants libraries using strategies like protein surface topography (PST) [151,154,155] and the Poisson–Boltzmann or generalized Born and surface area continuum solvation (MM/PBSA and MM/GBSA) energy functions [134,156], and virtual screening software like DockoMatic [131,157] were developed with automated peptide analog creation.

**Table 5:** The summary of published ion channel modulators and the corresponding mammalian targets.

Modulator Name	Gene Superfamily	Cysteine Framework	Pharmacological Family	Function	Target	Refs.
MVIIA	O1	VI/VII	ω-conotoxin	inhibitor	Ca_V_2.1, Ca_V_2.2	[158,159]
TxVIA	O1	VI/VII	δ-conotoxin	activator/inhibitor	Ca_V_3.1, Ca_V_3.2	[127]
CIIIA	M	III	μ-conotoxin	inhibitor	Na_V_ TTX-R	[160]
CnIIIA		III	μ-conotoxin	inhibitor	Na_V_ TTX-R	[160]
GIIIA	M	III	μ-conotoxin	inhibitor	Na_V_1.4	[161,162]
PIIIA	M	III	μ-conotoxin	inhibitor	Na_V_1.4, NaChBac, Na_V_Sp1, Na_V_Ab, K_V_1.1, K_V_1.6	[113,121,128,163]
SIIIA	M	III	μ-conotoxin	inhibitor	Na_V_1.2, Na_V_1.4, Na_V_1.6, K_V_1.6	[113,164]
SxIIIC		III	μ-conotoxin	inhibitor	Na_V_1.7	[165]
KIIIA	M	III	μ-conotoxin	inhibitor	Na_V_1.2, Na_V_1.3, Na_V_1.4, Na_V_1.5, Na_V_1.6, Na_V_1.7, Na_V_ TTX-R	[111,166,167]
PVIIA	O1	VI/VII	κ-conotoxin	inhibitor	K_V_ Shaker	[168]
RIIIJ	M	III	κ-conotoxin	inhibitor	K_V_1.1, K_V_1.2, K_V_1.6	[169]
RIIIK	M	III	κ-conotoxin	inhibitor	TSha1 Kⴙ channels	[170]
AuIB	A	I	α-conotoxin	inhibitor	α3β4	[171]
PnIA	A	I	α-conotoxin	inhibitor	α7, α7-5HT3 chimaera	[172]
MII	A	I	α-conotoxin	inhibitor	α3β2, α6β2	[173]
Vc1.1		I	α-conotoxin	inhibitor	α9α10	[149]
ImI	A	I	α-conotoxin	inhibitor	α3β4, α7, α7-5HT3 chimaera	[172,174,175]
ImII	A	I	α-conotoxin	inhibitor	α7, α7-5HT3 chimaera	[175]
EpI	A	I	α-conotoxin	inhibitor	α3β4, α7	[176]
GIC	A	I	α-conotoxin	inhibitor	α3β2	[30]
GID	A	I	α-conotoxin	inhibitor	α4β2	[135]
TxIA	A	I	α-conotoxin	inhibitor	α4β2	[177]
TxID		I	α-conotoxin	inhibitor	α3β4, α6/α3β4	[140]
RgIA	A	I	α-conotoxin	inhibitor	α9α10	[147]
MrIC	A	I	α-conotoxin	activator	α7	[178]

## 5. Discussion

Conotoxins are undeniably valuable pharmacological molecular probes and hold high therapeutic potential for neurological disorders and diseases in the spotlight of marine drug development. The development of in silico techniques for conotoxin studies has overcome the shortcomings of traditional techniques (time-consuming, expensive, and low-accuracy), and facilitated conotoxin classification, mechanism of action and structure–activity investigations as well as novel drug discovery with high-sensitivity, high-specificity and high-accuracy. This review mainly focuses on the current progress in in silico conotoxin classification and discovery, de novo conotoxin drug design, conotoxin–ion channel interaction investigation, and virtual conotoxin drug screening.

Snail multi-omics, integrating with genomics, transcriptomics, proteomics, and bioinformatics tools, has made conspicuous contributions to novel peptide toxin discovery and faced the challenges of handling non-conservative models [179]. The website of ConoServer has hitherto described 3073 nucleotide sequences, 8523 protein sequences, and 246 structures, covering 90 species, 123 species, and 48 species of cone snails, respectively (October 2024). Thanks to the introduction of advanced experimental methods, particularly Cryo-EM, the number and quality of channel/receptor protein structures increasingly improved, providing more access to homology models and creating conditions for computer-aided pharmacological studies. In addition to experimental approaches, AI advancements also highlight their role in accelerating drug discovery through accurately predicting protein structures. AlphaFold (1–3), as a representative, allows rapid structure prediction, even in the absence of experimentally resolved structures. With access to protein structures, molecular docking, which is indispensable in in silico conotoxin drug discovery, is now more accessible and reliable. Except for the more specific peptide docking tools like the RosettaDock, ToxDock and DockoMatic that we mentioned above, there are more generally available docking suites include DOCK, AutoDock, GOLD, FlexX, GLIDE, MOE-Dock, and Surflex-Dock, and web-based servers like PatchDock, HEX, and HADDOCK. In the investigation of binding modes, docking approaches provided visual mechanisms and dynamics results of conotoxin-target binding, such as binding affinities, binding energies, binding sites, and the key residues involving salt bridges, hydrogen bonds, and electrostatic effects.

Unlike small molecules, venom peptides have substantial barriers, such as a lack of solved structures and comparably large size [135], that must be addressed when considering in silico procedures. So far, more than 97% of conotoxin sequences are short of 3D structure and functional information [26,50,97,180]. What is more, the extreme sequence diversity and conformational flexibility are also complicating the predicting of peptide toxin bioactivities and functions from sequence data for homology-based machine learning techniques [181,182,183]. In the case of conotoxins, their structure prediction is further complicated by their extensive PTMs, as the current predicting tools, even AlphaFold, are less effective in PTM-involved predictions due to the lack of robust datasets as well as the complexity of how PTMs alter folding and dynamics. Likewise, there are limited experimental 3D structures of channels/receptors, especially nAChRs and human VGSCs, which slowed down the mechanism studies and set barriers for the virtual screening of active conotoxins. The low sequence similarity between templates and nAChRs also suggests that it is probably problematic for the virtual screening program to utilize the homology models of AChBP as the templates to construct heteromeric nAChRs [135,184]. Moreover, the docking poses and scores are overly dependent on pioneer knowledge and the input conformation of ligands by operators, resulting in significant differences caused by subtle variation [88]. Although machine learning can identify potential conotoxin candidates, it is nonnegligible that the theoretical model has its limitations such as the disagreement of the predicted results from the different in silico predictors and, therefore, should be compared with experimental models if possible. In silico hits should be subjected to in vitro and subsequently in vivo settings for their therapeutic potential [185,186].

The future holds some promising research directions, which not only compensate for the limitations of the current conditions but also blaze new trails in the field of in silico conotoxin studies. To start with, there is abundant room for further progress in enriching conotoxin sequences and PTM diversity using transcriptome sequencing of more samples at various development stages of cone snails and other tissues apart from the traditional venom ducts [46,97]. The advent of a new powerful algorithm is expected with a more sophisticated version of either a molecular docking or a molecular dynamics simulation program, to rapidly and accurately predict the binding modes of the conotoxins bound to their channels/receptors, providing persuasive explanations of the underlying mechanisms of modulation [8]. For better model construction, advanced feature selection techniques, such as minimal-redundancy–maximal-relevance (mRMR), principal component analysis (PCA), and analysis of variance (ANOVA), could be combined to overcome the difficulties from high-dimension features and extract the key features that contribute to model accuracy. On the other hand, constructing high-quality and reliable benchmark datasets that are diverse and representative of the conotoxin sequences could ensure the models are trained on a wide range of samples and can overcome the peptide conformational flexibility [187]. To some extent, the current studies are limited to specific subtypes of ion channels or receptors; it is probably worth a more extensive investigation on the discovery of conotoxin modulators for a larger scope of human protein targets if possible [188,189]. From a short-term perspective, the application of AlphaFold in in silico conotoxin studies could be expanded from protein structure prediction to the prediction of conotoxin-target joints to have a direct idea of their interactions. As we gradually refine the theoretical models and harness the power of in silico conotoxin studies more comprehensively, we will be able to precisely design conotoxins that can modulate disease-relevant targets with high selectivity and potency, and the prediction of conotoxin drug candidates will be brought to the next level.

## Figures and Tables

**Table 1:** Top 10 keyword co-occurrence of in silico conotoxin studies (2004–2024).

Keyword	Occurrence	Total Link Strength
Conotoxin	64	290
Crystal structure	50	265
Conotoxin superfamily	42	274
Molecular dynamics	40	160
Support vector machine	26	178
Binding	25	122
α-conotoxin	23	139
Protein	23	106
Ensemble classifier	22	156
Amino acid composition	22	133

**Table 2:** Summary of cysteine frameworks, with Cystine pattern and number of cysteines [54,55]. Data derives from ConoServer (https://www.conoserver.org/), a database providing standardized annotations of conopeptides.

Framework	Cystine Pattern	Cysteines
I	CC–C–C	4
II	CCC–C–C–C	6
III	CC–C–C–CC	6
IV	CC–C–C–C–C	6
V	CC–CC	4
VI/VII	C–C–CC–C–C	6
VIII	C–C–C–C–C–C–C–C–C–C	10
IX	C–C–C–C–C–C	6
X	CC–C–C	4
XI	C–C–CC–CC–C–C	8
XII	C–C–C–C–CC–C–C	8
XIII	C–C–C–CC–C–C–C	8
XIV	C–C–C–C	4
XV	C–C–CC–C–C–C–C	8
XVI	C–C–CC	4
XVII	C–C–CC–C–CC–C	8
XVIII	C–C–CC–CC	6
XIX	C–C–C–CCC–C–C–C–C	10
XX	C–CC–C–CC–C–C–C–C	10
XXI	CC–C–C–C–CC–C–C–C	10
XXII	C–C–C–C–C–C–C–C	8
XXIII	C–C–C–CC–C	6
XXIV	C–CC–C	4
XXV	C–C–C–C–CC	6
XXVI	C–C–C–C–CC–CC	8
XXVII	C–C–C–CCC–C–C	8
XXVIII	C–C–C–CC–C–C–C–C–C	10
XXIX	CCC–C–CC–C–C	8
XXX	C–C–CCC–C–C–C–CC	10
XXXII	C–CC–C–C–C	6
XXXIII	C–C–C–C–C–C–C–C–C–C–C–C	12

**Table 3:** Summary of pharmacological families, definition and their representatives [54,55]. Data derived from ConoServer (https://www.conoserver.org/) (accessed on 1 October 2024).

Family	Definition	Representative
α (alpha)	Nicotinic acetylcholine receptors (nAChR)	GI
γ (gamma)	Neuronal pacemaker cation currents (inward cation current)	PnVIIA, TxVIIA
δ (delta)	Voltage-gated Na channels (agonist, delay inactivation)	TxVIA
ε (epsilon)	Presynaptic Ca channels or G protein-coupled presynaptic receptors	TxVA
ι (iota)	Voltage-gated Na channels (agonist, no delayed inactivation)	RXIA
κ (kappa)	Voltage-gated K channels (blocker)	PVIIA
μ (mu)	Voltage-gated Na channels (antagonist, blocker)	GIIIA
ρ (rho)	Alpha1-adrenoceptors (GPCR)	TIA
σ (sigma)	Serotonin-gated ion channels 5-HT3	GVIIIA
τ (tau)	Somatostatin receptor	CnVA
χ (chi)	Neuronal noradrenaline transporter	MrIA, CMrVIA

**Table 4:** Accuracy comparison of different prediction algorithms for conotoxin–ion channel prediction.

Method	Overall Accuracy (%)	Average Accuracy	Reference
F-score-SVM	94.6	94.2	[69]
AVC-SVM	91.98	92.17	[104]
IonchanPred 2.0	92.6	87.7	[105]
RBF network	89.3	89.7	[106]
iCTX-Type	91.1	90.3	[107]
ICTCPred	-	91.0	[108]

## Data Availability

Bibliometric data are contained within article.
